# Assembling Complex Macromolecules and Self-Organizations of Biological Relevance with Cu(I)-Catalyzed Azide-Alkyne, Thio-Bromo, and TERMINI Double “Click” Reactions †

**DOI:** 10.3390/polym15051075

**Published:** 2023-02-21

**Authors:** Adrian Moreno, Gerard Lligadas, Jasper Adamson, Devendra S. Maurya, Virgil Percec

**Affiliations:** 1 Laboratory of Sustainable Polymers, Department of Analytical Chemistry and Organic Chemistry, University Rovira i Virgili, 43007 Tarragona, Spain; 2 Roy & Diana Vagelos Laboratories, Department of Chemistry, University of Pennsylvania, Philadelphia, PA 19104-6323, USA; 3 Chemical Physics Laboratory, National Institute of Chemical Physics and Biophysics, Akadeemia tee 23, 12618 Tallinn, Estonia

**Keywords:** click reactions, Cu(I)-catalyzed azide-alkyne click, thio-bromo click, termini dual click, dendrimersomes, glycodendrimersomes, dendrimers, biological membranes, modular-orthogonal methodology

## Abstract

In 2022, the Nobel Prize in Chemistry was awarded to Bertozzi, Meldal, and Sharpless “for the development of click chemistry and biorthogonal chemistry”. Since 2001, when the concept of click chemistry was advanced by Sharpless laboratory, synthetic chemists started to envision click reactions as the preferred choice of synthetic methodology employed to create new functions. This brief perspective will summarize research performed in our laboratories with the classic Cu(I)-catalyzed azide-alkyne click (CuAAC) reaction elaborated by Meldal and Sharpless, with the thio-bromo click (TBC) and with the less-used, irreversible TERminator Multifunctional INItiator (TERMINI) dual click (TBC) reactions, the last two elaborated in our laboratory. These click reactions will be used to assemble, by accelerated modular-orthogonal methodologies, complex macromolecules and self-organizations of biological relevance. Self-assembling amphiphilic Janus dendrimers and Janus glycodendrimers together with their biological membrane mimics known as dendrimersomes and glycodendrimersomes as well as simple methodologies to assemble macromolecules with perfect and complex architecture such as dendrimers from commercial monomers and building blocks will be discussed. This perspective is dedicated to the 75th anniversary of Professor Bogdan C. Simionescu, the son of my (VP) Ph.D. mentor, Professor Cristofor I. Simionescu, who as his father, took both science and science administration in his hands, and dedicated his life to handling them in a tandem way, to their best.

## 1. Introduction

In the late 1990s, the golden dream of chemists to perform chemical transformations in living systems raised interest in a set of chemical reactions that are selective without interfering with native biochemical processes. In this context, Bertozzi, Raines, and Kiessling laboratories transformed the classic Staudinger reaction between triarylphosphines and azides into bioorthogonal Staudinger ligation [1,2,3,4,5]. This reaction allowed for the first time to perform chemistry on cultured cells and in living animals. At the same time Sharpless laboratory [6] introduced the concept of click chemistry as a “set of powerful, highly reliable, and selective reactions for the rapid synthesis of useful new compounds and combinatorial libraries”. Sharpless pointed out the following requirements for click reactions: modularity, wide-scope, very high yield, stereospecificity (but not necessarily enantioselectivity), and simple product isolation (separation from harmless by-products by non-chromatographic methods). In addition to these criteria, these reactions should proceed using simple reaction conditions (solvent free or solvents like water) including simply accessible starting materials, and the final product has to be stable under physiological conditions. Sharpless laboratory pointed out also to a group of old reactions belonging to the class of click reactions. Since 2002, the Cu(I)-catalyzed regioselective “ligation” of azides with alkynes—i.e., Cu(I)-catalyzed azide-alkyne cycloaddition (CuAAC)—reported independently by Meldal [7] and Sharpless [8] laboratories, inspired from Huisgen [9] corresponding cycloaddition became the classic “click” reaction. However, the requisite use of a transition metal catalyst prevented trajectories of bioorthogonal and click chemistries from converging until the development of metal-free “click” strategies came to the scene. For example, the strain-promoted azide-alkyne cycloaddition proposed by Bertozzi laboratory allowed the reaction to proceed quickly in biological systems and without living cell toxicity [10]. The Nobel Prize in Chemistry for the year 2022 was awarded to Bertozzi, Meldal, and Sharpless “for the development of click chemistry and biorthogonal chemistry”. Today, the “click” toolbox available for chemists, material scientists, and biologists is broad and includes various types of 1,3-dipolar cycloadditions, triazolinedione-based reactions, oxime ligations, Diels–Alder cycloadditions, thiol-based couplings, sulfur(VI)–fluoride exchange reactions, and many other. This brief personal Perspective summarizes research performed mostly in our laboratories with modular-orthogonal strategies based on CuAAC [11,12], thio-bromo click (TBC) [13,14,15], and irreversible TERminator Multifunctional INItiator (TERMINI) double click (TDC) [16,17] reactions to assemble complex macromolecules and self-organizations of biological relevance.

## 2. A Brief Discussion of the Development of CuAAC, TBC, and TDC Concepts

**Figure 1:**
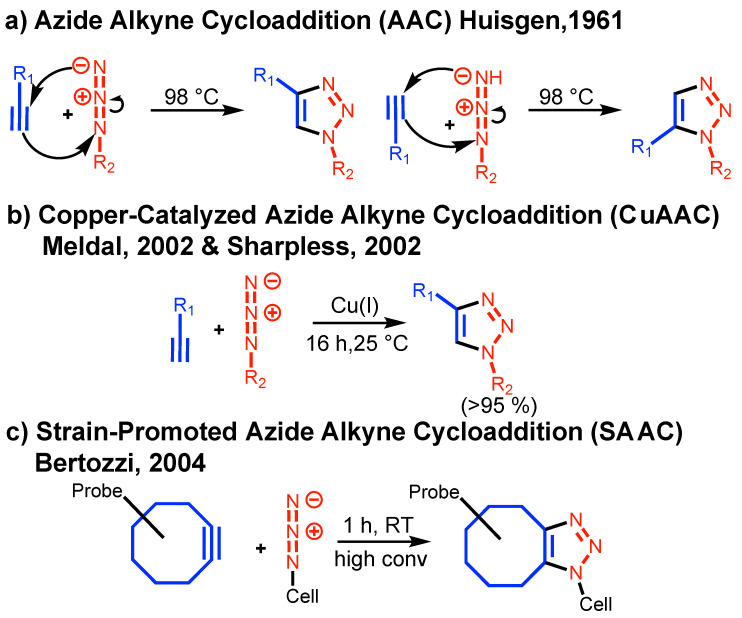
The concepts of AAC, CuAAC, and SAAC.

The historical developments of azide-alkyne cycloaddition (AAC) reaction, its Cu(I)-catalyzed (CuAAC) and strain-promoted (SAAC) versions are summarized in Figure 1.

Figure 1a outlines the original azide-alkyne cycloaddition elaborated by Huisgen [9] and named as one of the first click reactions by Sharpless [6]. This reaction requires either high reaction temperature or long reaction time to reach high conversion and lacks regiospecificity. In 2002, Meldal [7] and Sharpless [8] laboratories developed the Cu(I)-catalyzed AAC (CuAAC) to provide regiospecificity, high conversion and short reaction times at room temperature. In 2004, Bertozzi laboratory incorporated strain in the structure of the alkyne to generate strain-promoted [3 + 2] azide-alkyne cycloaddition (SAAC) (Figure 1c) [10]. SAAC can be performed at room temperature in vivo but the cycloaddition loses regiospecificity. The following two review articles discussing in more details these developments are recommended [18,19]. CuAAC became the classic click methodology for in vitro experiments while SAAC became the classic biorthogonal methodology for in vivo experiments. Numerous review articles covering the explosion of developments both for the in vitro and in vivo developments of/or based on click chemistry are available [18,19,20,21,22,23,24].

**Figure 2:**
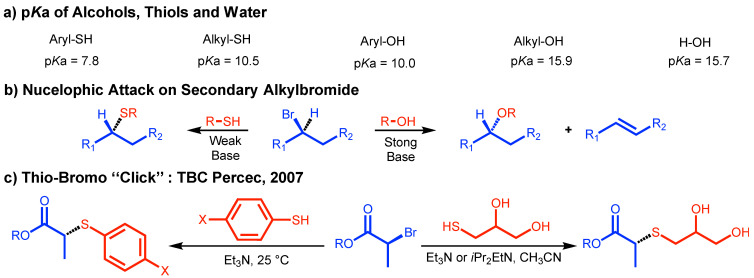
The concept of TBC.

Figure 2 outlines the development of thio-bromo click (TBC) reaction. In 2007, one of our laboratories was working on the elaboration of SET-LRP an ultrafast living radical polymerization method for acrylates, methacrylates, styrenes, and vinyl chloride producing ultrahigh molar mass polymers at room temperature. SET-LRP was reported by our laboratory in 2006 [25,26]. Our hypothesis for these very high molar mass polymers was a very low degree of bimolecular termination and therefore, very high chain end functionality for the resulting polymers. Since acrylates have the highest rate of polymerization, we had to develop a method for the quantitative determination of the polymer chain end functionality. The chain end resulted from the SET-LRP of acrylates was a secondary alkyl halide. The simplest functionalization of a secondary alkyl halide would be by an S_N_2 reaction. However, in the presence of alkoxy nucleophiles, secondary alkyl halides will undergo both E2 and S_N_2 reactions (Figure 2a,b). A softer thiolate nucleophile would have the chance to undergo only the S_N_2 reaction required for this process. If this reaction would work, it would provide access to a simple functionalization of the chain end(s) since alkyl thiols and thiophenols have a much lower pKa than the corresponding precursors to the oxygen nucleophiles (Figure 1a). The first attempt to functionalize the chain end(s) of polyacrylates with thiophenol deprotonated by Et_3_N in acetonitrile was a great success [13] that paved the way to the very simple and versatile thio-bromo click (TBC) reaction employed to determine the chain end functionality of polyacrylates and to assemble complex macromolecules and self-organizations. Review articles dedicated entirely to TBC are not available. However, TBC based applications are discussed in several general review articles [23,26,27,28].

**Figure 3:**
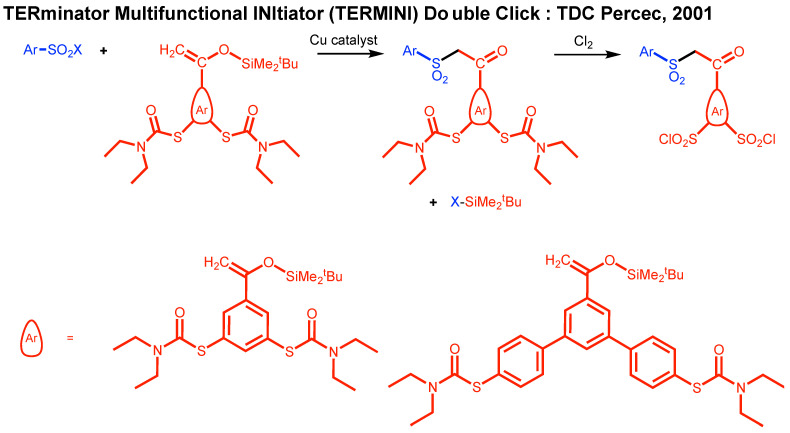
The concept of TDC.

Figure 3 summarizes the TERminator Multifunctional INItiator (TERMINI) double click (TDC) chemistry concept. The TDC chemistry started to be elaborated in 1998 when our laboratory demonstrated that arenesulfonyl halides are a universal class of functional initiators for metal-catalyzed living radical polymerization of styrene(s), methacrylates, and acrylates [29]. In the same paper, we demonstrated that quantitative addition of arylsulfonyl radicals to styrene and methyl methacylate takes place in about 5 min and under proper reaction conditions, is not accompanied by polymerization, allowing determination the rate constant of initiation for several different monomers for the first time. In 2001, the addition of arylsulfonyl radicals to an even more reactive 1,1-disubstituted vinyl monomer, an enol of an aryl monomer containing two masked sulfonyl halides has been shown to occur by transforming the enol into a keto and thus self-interrupting a radical reaction/polymerization process. Subsequently the masked diethyldithiocarbamate groups were transformed quantitatively in about 2 min in the presence of Cl_2_ into the corresponding arylsulfonyl halide initiators (Figure 3) [16]. This TDC process was immediately applied to the synthesis of dendrimers from conventional commercial monomers with a large diversity of TERMINI and multifunctional sulfonyl chloride initiators [17,30,31,32]. Brief review articles summarizing the role of TDC in the development of SET-LRP and of polymers with complex architecture are available [26,27,28].

## 3. Dendrimersomes and Glycodendrimersomes as Mimics of Biological Membranes

**Figure 4:**
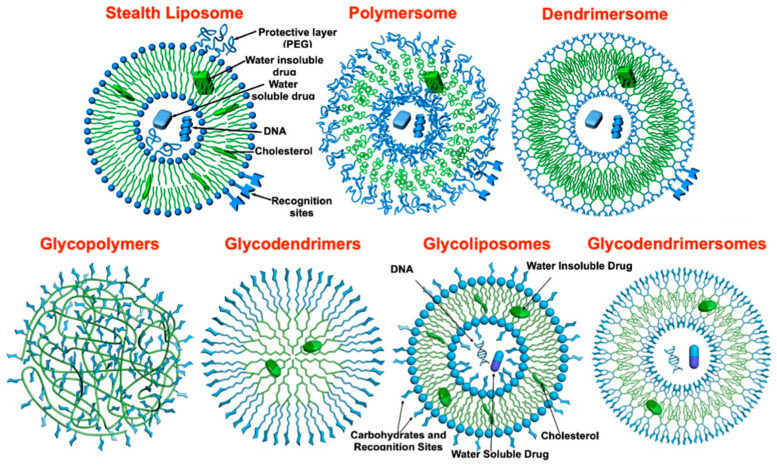
The development of Stealth liposomes, polymersomes, dendrimersomes, glycopolymers, glycodendrimers, glycoliposomes, and glycodendrimersomes.

Figure 4 summarizes the concept of dendrimersomes and glycodendrimersomes as mimics of biological membranes. In 1964, Bangham laboratory reported that natural phospholipids self-assemble into liposome that are however unstable [33]. Increased stability of liposomes including in vivo was accomplished by co-assembly with PEG-conjugated phospholipids and cholesterol to create Stealth Liposomes that are currently used in drug delivery [34,35]. The second approach to increased stability of vesicles was obtained by self-assembly of amphiphilic block copolymers [36]. The resulting vesicles—named polymersomes—are stable; exhibit excellent mechanical properties, but the thickness of their bilayer is much wider than that of the cell membranes; and their building blocks are polydisperse, even when they are prepared by living polymerization methodologies. In 2010, our laboratory reported that monodisperse amphiphilic Janus dendrimers self-assemble into vesicles named dendrimersomes, that are stable, exhibit excellent mechanical properties, and their bilayer thickness is identical with that of the cell membranes. In addition, Janus dendrimers self-assemble into dendrimersomes with predictable dimensions by simple injection from their ethanol solution in water or buffer [11]. The multivalency of the glycan of biological membranes was first mimicked with glycopolymers [37], followed by glycodendrimers [38] and glycoliposomes [39]. Glycopolymers were the first mimics of the glycan of biological membranes. They are easy to synthesize but most of the carbohydrates are part of the inner structure of the random-coil conformation of the glycopolymers and therefore, the exact value of the multivalency on the surface of glycopolymers is unknown. Glycodendrimers have a good control of the carbohydrate multivalency but their synthesis is very difficult. Glycoliposomes are made by co-assembly of phospholipids with sugar conjugated lipids and this process limits our knowledge of their exterior multivalency. In 2013, by screening through numerous libraries, our laboratory elaborated the synthesis of amphiphilic Janus dendrimers whose self-assembly provides very precise and predictable size vesicles named glycodendrimersomes [12]. Just like Janus dendrimers, Janus glycodendrimers self-assemble in water or in buffer by simple injection of their ethanol or THF solution into monodisperse glycodendrimersomes with predictable dimensions. CuAAC was used in their modular orthogonal synthesis. Several review articles discuss their synthesis, self-assembly of dendrimersomes and glycodendrimersomes, and their biological activity in interaction with sugar binding proteins known as lectins [23].

## 4. Perfecting SET-LRP with the Aid of TBC

**Figure 5:**
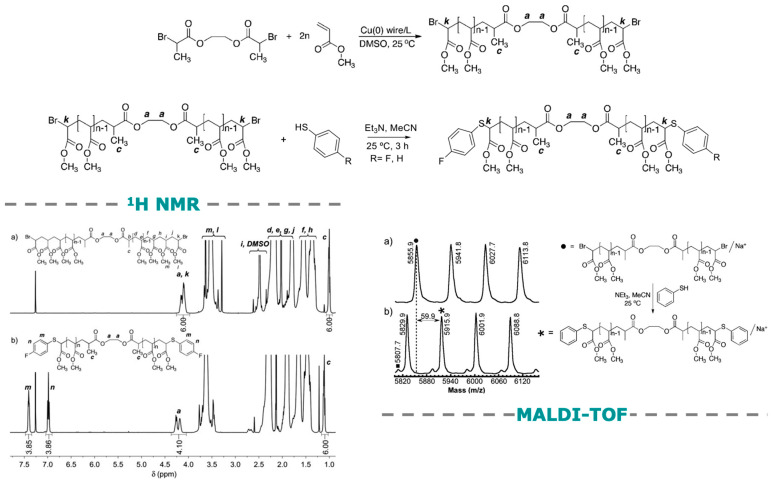
Structural analysis of polyacrylate chain ends by TBC.

A simple and rapid method for polymer chain end(s) analysis by a combination of accurate and accessible analytical methods is required in order to perfect the structure of the polymers synthesized by living radical polymerization reaction under different reaction conditions. A combination of NMR and MALDI-TOF is considered by us to provide such a methodology. The major requirement for such a methodology is based on a rapid and quantitative organic reaction that transforms the structure of the polymer chain end(s) from its native functional group into a new functional group. Polyacrylates synthesized by SET-LRP contain a secondary alkyl bromide native functional group. In 2007, we considered that TBC could potentially provide this methodology if the secondary bromide group could be replaced with an aromatic thioether group (Figure 2) by an S_N_2 mechanism in the complete absence of an E2 reaction. Thiophenol and *p*-fluorothiophenol would be suitable candidates for this reaction since their suitable pKa would provide their transformation into a soft nucleophile in situ by using mild bases such as NEt_3_ in the low boiling polar solvent acetonitrile. In addition, the resulting new thiophenolate chain-end would exhibit ^1^H-NMR resonance that would not overlap with the structure of the polyacrylate, but would be able to be integrated with the structure of the initiator rest and of the parent bromide native chain end. Figure 5 outlines this TBC chain end analysis method reported by our laboratory in 2007 [13]. The TBC strategy outlined in Figure 2 and Figure 5 became a routine method employed to analyze the structure of polymer chain-ends both in our laboratory as well as in other laboratories and to construct polymers with complex architecture [13,14,15,40,41,42,43,44,45,46,47,48,49,50,51,52,53,54,55,56,57,58,59,60,61,62,63,64,65,66,67,68,69,70,71,72,73,74,75,76,77,78,79,80,81,82,83,84,85,86,87,88,89,90,91,92,93,94,95,96,97,98,99,100,101,102]. This method was expanded by our and other laboratories to the construction of polymers with complex architecture and functionality including dendrimers.

## 5. Divergent Synthesis of Dendrimers by TBC

**Figure 6:**
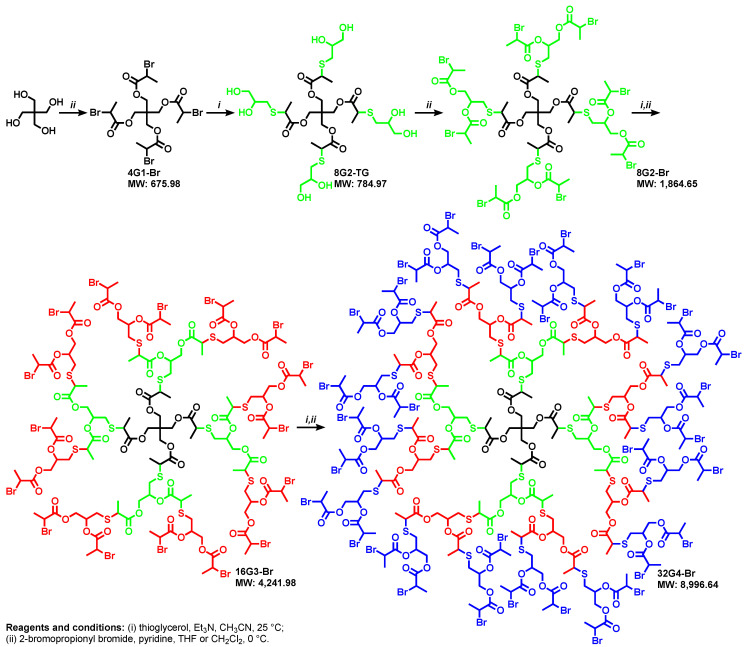
Synthesis of G1 to G4 dendrimers by TBC [14]. Reproduced with permission from Ref. [14]; Copyright 2009 John Wiley & Sons, Inc.

The difference between the pKa of alcohols and thiols outlined in Figure 2a was used to employ bifunctional thiol-alcohol organic compounds such as thioglycerol in the synthesis of complex monodisperse macromolecules by the TBC chemistry as outlined in the right part of Figure 2c. In the presence of Et_3_N or iPr_2_EtN as base in acetonitrile the thiol groups is deprotonated and therefore, is transformed into a soft nucleophile and very weak base, while the alcohol groups that are strong bases when deprotonated are not deprotonated. The difference in nucleophilicity and basicity between the deprotonated thiol and the non-deprotonated alcohol groups make the alcohol groups be inert and inactive during this TBC reaction in which the thioglycerol group incorporates the AB2 branching point. This combination of reactivity of the thioglycerol was employed by one of our laboratories to develop a new methodology for the divergent synthesis of dendrimers (Figure 6) [14].

## 6. Divergent Synthesis of Dendritic Macromolecules from Commercial Monomers by TBC

**Figure 7:**
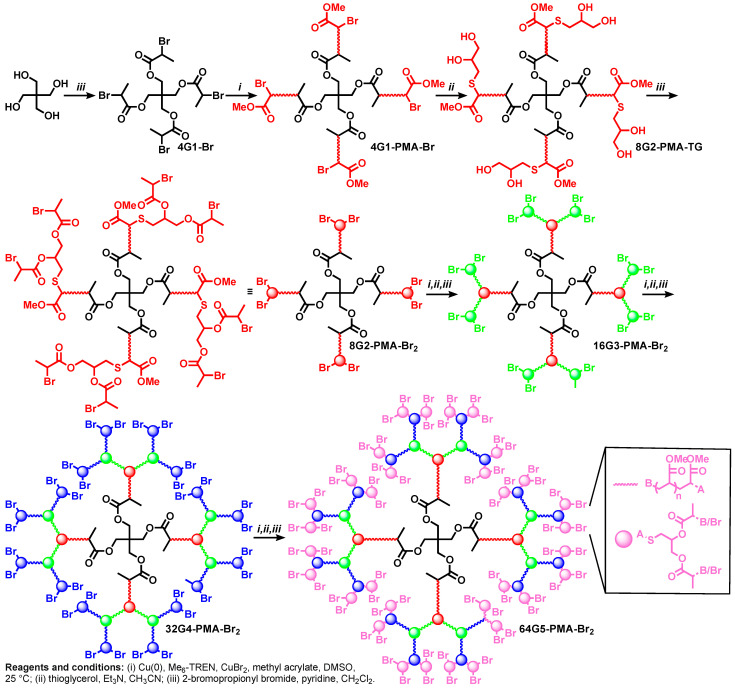
Synthesis of dendritic polyacrylates by TBC combined with SET-LRP [15]. Reproduced with permission from Ref. [15]; Copyright 2009 John Wiley & Sons, Inc.

An additional divergent methodology for the synthesis of dendrimers based on and TBC (Figure 2 and Figure 6) was elaborated by combining the thioglycerol and SET-LRP of commercial monomers. Figure 7 illustrates this new methodology for the assembly of dendritic macromolecules from methyl acrylate [15]. This method can be applied to the synthesis of dendritic macromolecules from any commercial acrylate or acrylamide monomers that does not contain alcohol or amine groups. Alternative methods for the synthesis of dendrimers by TBC chemistry were elaborated [103,104,105].

## 7. Modular-Orthogonal Assembly of Amphiphilic Janus Dendrimers by TBC

**Figure 8:**
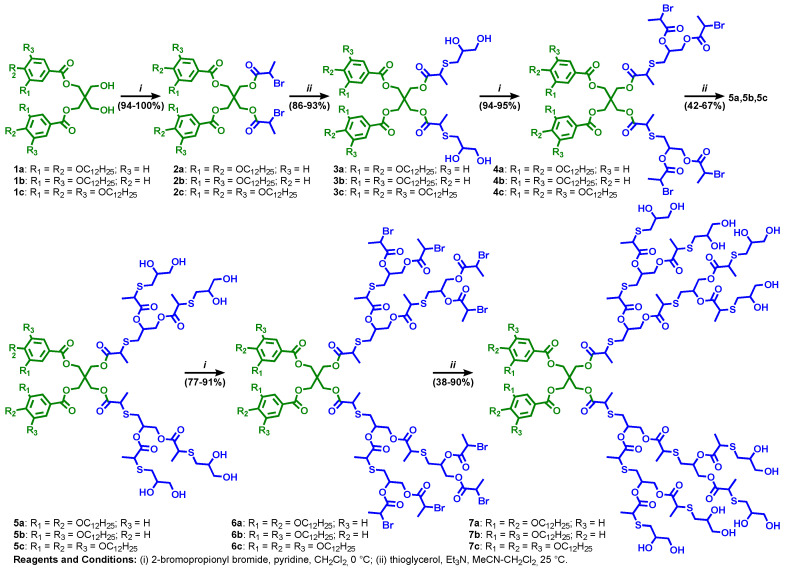
Synthesis of thioglycerol-benzylether amphiphilic Janus dendrimers by TBC [11]. Reproduced from Ref. [11] with permission from AAAS.

**Figure 9:**
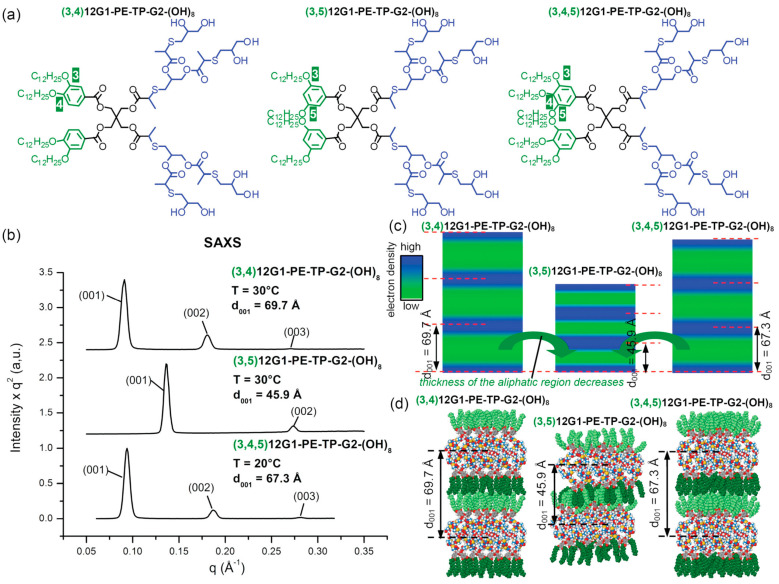
Chemical structures (**a**) with the corresponding SAXS data for the lamellar phases of the indicated library of three JDs synthesized by TBC (**b**). X-ray data and reconstructed electron density maps (**c**) illustrating the change in thickness of the layers from less interdigitated (3,4)12G1-X and (3,4,5)12G1-X to more interdigitated (3,5)12G1-X JDs (**d**) [121]. Reprinted with permission from Ref. [121]; Copyright 2011 American Chemical Society.

**Figure 10:**
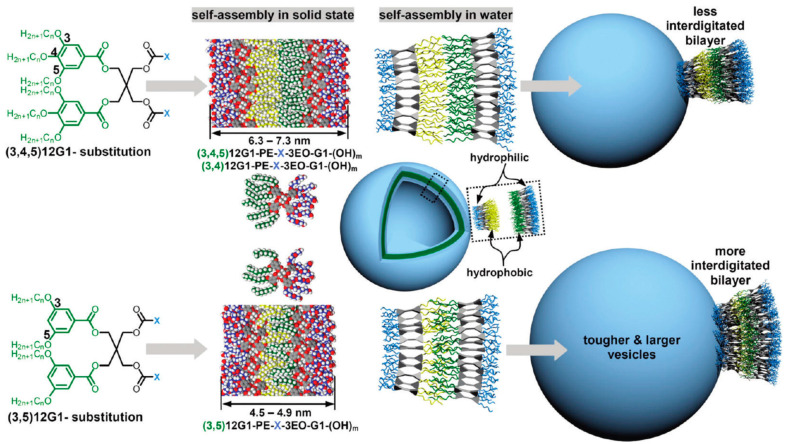
Illustration of the self-assembly of twin–twin JDs into DSs. For clarity, the alkyl chains of the inner and outer leaflets of the bilayer are depicted in yellow and green, respectively [121]. Reprinted with permission from Ref. [121]; Copyright 2011 American Chemical Society.

Amphiphilic Janus dendrimers that self-assemble dendrimersomes were elaborated by a diversity of synthetic methodologies [11,23] to self-assemble unilamellar and multilamellar onion-like assemblies [106,107,108,109,110,111,112,113,114,115,116,117,118,119,120]. A schematic synthesis of amphiphilic Janus dendrimers by employing the thioglycerol TBC outlined in Figure 2c demonstrates the capabilities of this strategy (Figure 8). The hydrophilic part of the Janus dendrimers is determined by the generation number of the hydrophilic dendron prepared by TBC chemistry while the hydrophobic part is determined by the 3,5-, 3,4-, or 3,4,5- substitution pattern of the corresponding phenolic acid precursor. The 3,5-disubstituted hydrophobic fragments interdigitate in their hydrophobic part providing a thinner bilayer. The 3,4- and 3,4,5- substitution patterns do not interdigitate and therefore, provide a wider bilayer. This information is obtained from the X-ray analysis of the lamellar structure (Figure 9). When transplanted to water or buffer phase, the mechanism outlined in Figure 9 from bulk state provides access to a methodology to predict with great accuracy the size of the dendrimersome assembled in water or buffer (Figure 10) [121].

## 8. Modular-Orthogonal CuAAC Synthesis of Amphiphilic Janus Glycodendrimers

**Figure 11:**
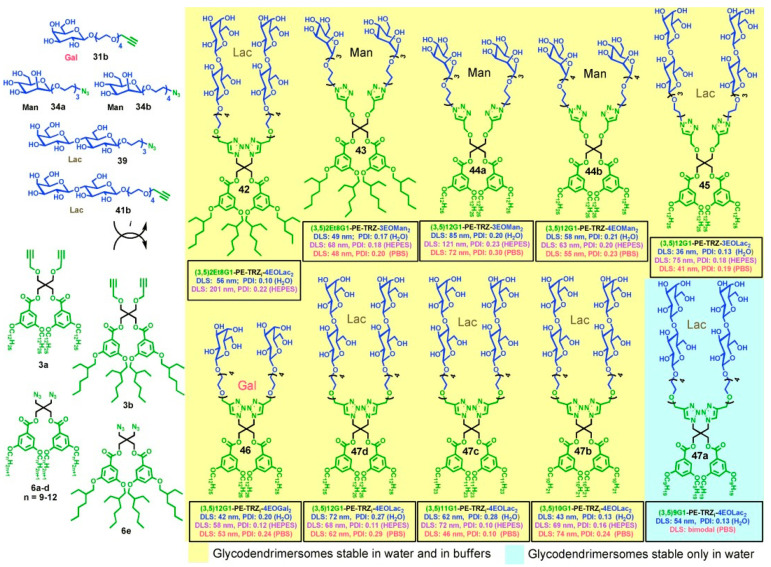
Examples of modular-orthogonal synthesis of twin–twin JGDs by CuAAC. Reagents and conditions: (i) CuSO_4_·5H_2_O, sodium ascorbate, THF/water (25 °C) [122]. Reprinted with permission from Ref. [122]; Copyright 2009 John Wiley & Sons, Inc.

An accelerated modular-orthogonal CuAAC synthesis of amphiphilic single–single, twin–twin, and twin–mixed Janus glycodendrimers containing representative plant, bacteria, and human carbohydrates in their hydrophilic part that self-assemble by simple injection in water or buffer into glycodendrimersomes was elaborated in our laboratory in 2013 [12] and developed in additional publications [122,123,124,125,126,127,128,129,130,131,132,133,134]. Single–single stands for a combination of single hydrophilic with single hydrophobic dendrons in the structure of the amphiphilic Janus dendrimer, twin–twin stands for two identical hydrophilic and two identical hydrophobic dendrons while twin–mixed represents a combination of twin hydrophobic and mixed hydrophilic. This definition will become more trivial as we will follow the Figures in which these structures will be discussed. Figure 11 outlines the modular-orthogonal synthesis of a library of twin–twin.

**Figure 12:**
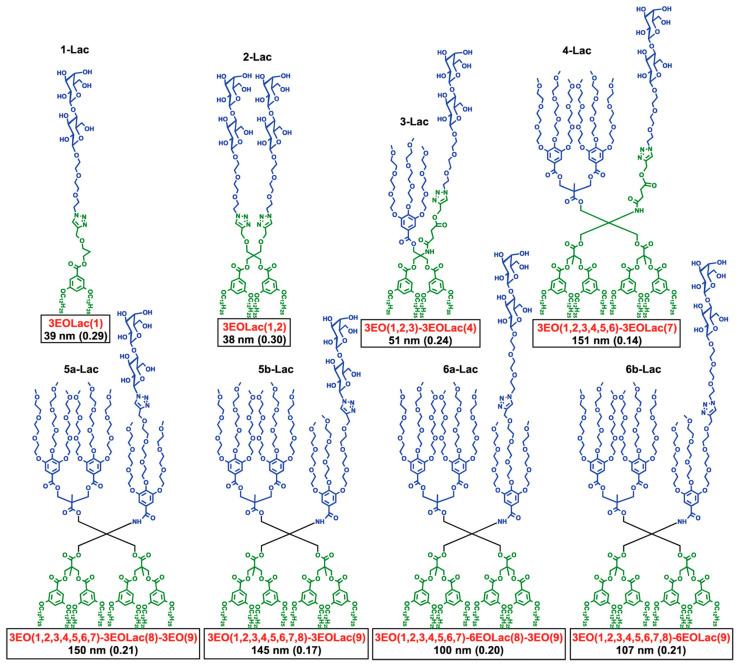
Summary of amphiphilic JDs with sequence-defined arrangement of Lac in the hydrophilic segment exhibiting different density. The diameter (DDLS, in nm) and polydispersity (in parentheses) were determined by DLS at 0.1 mM of Lac in PBS [125]. Reprinted with permission from Ref. [125]; Copyright 2015 American Chemical Society.

**Figure 13:**
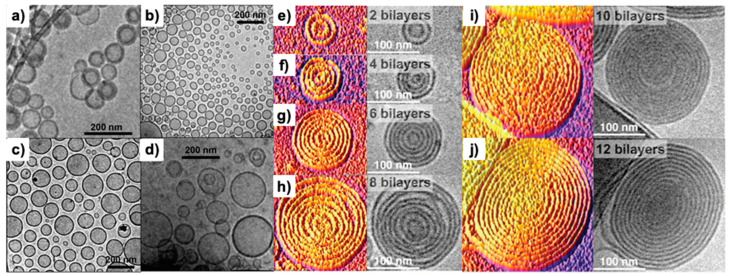
Representative cryo-TEM images of GDSs self-assembled from twin–twin JGDs (**a**–**d**). Representative cryo-TEM images of onion-like GDSs self-assembled from JGDs and their 3D intensity-plotting images with different numbers of bilayers and diameters at 0.1 mM in HEPES (**e**–**j**) [12,126]. Reproduced with permission from Refs. [12,126]; copyright 2013 American Chemical Society and 2016 National Academy of Sciences USA.

Janus glycodendrimers by CuAAC chemistry. Twin hydrophobic fragments are equipped with azide or terminal alkyne groups while the corresponding carbohydrate libraries are conjugated to the complementary azide and alkyne groups required to create the modules employed for orthogonal CuAAC coupling. Similar methodologies were elaborated for libraries of single–single and twin–mixed Janus glycodendrimersomes. The primary structure of the hydrophilic and hydrophobic parts were modified until conditions were found to design amphiphilic Janus glycodendrimers that self-assemble and are stable in the buffer required to study the interaction between the glycan surface of the glycodendrimersomes with natural sugar-binding proteins specific for plant, bacterial, and human cells—known as lectins—and with synthetically modified and programmed lectins. One of the many questions we had to address was the density of sugars on the surface of our glycodendrimersome that provides the highest activity. Intuitively, the higher the density of sugars the higher is the multivalency of the glycan and therefore, a higher activity of binding to proteins is expected. This concept was studied by generating a library of Janus glycodendrimers that provides a variation in the concentration and sequence of sugars on the glycodendrimersome surface. Figure 12 provides an example of methodology that generates access to sequence and concentration dependence of the glycan. This concentration has to take into account the fact that glycodendrimersomes can be either unilamellar or onion-like multilamellar architectures (Figure 13).

**Figure 14:**
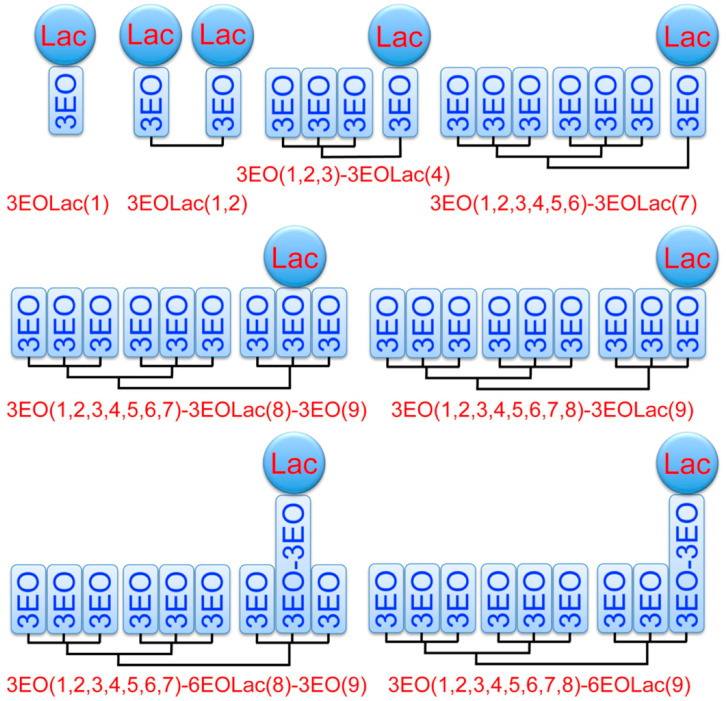
Programming biological activity with sequence-defined glycodendrimersomes. Summary of Lac-containing amphiphilic Janus dendrimers used for agglutination assays with galectin-8 (3EO = methoxytriethoxy group and Lac = d-lactose). The hydrophobic segments of these molecules, triazoles, and aromatic rings are omitted for clarity [125]. Reprinted with permission from Ref. [125]; Copyright 2015 American Chemical Society.

**Figure 15:**
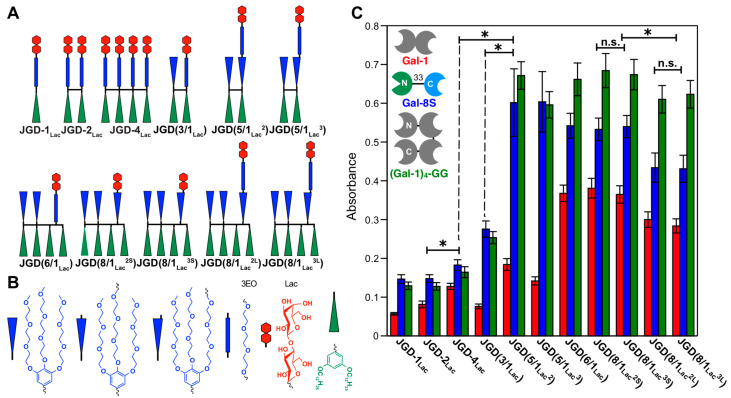
Encoding biological recognition in a bicomponent cell-membrane mimic. (**A**) Sequence-defined JGDs with different Lac density, sequence, and linker length. (**B**) Schematic representation of JGD building blocks. (**C**) Summary of aggregation assay data using GDSs from self-assembly of sequence-defined JGDs (Lac = 0.1 mM, 900 μL) with Gal-1 (1 mg·mL^−1^, 100 μL), Gal-8S (1 mg·mL^−1^, 100 μL), and (Gal-1)4–GG (1 mg·mL^−1^, 100 μL). Color codes for galectins: Gal-1, red; Gal-8S, blue; (Gal-1)4–GG, green. N and C represent the N and C termini of proteins. For selected examples symbols used for significant difference (*p* values by Student’s *t*-test) are: “n.s.” for *p* > 0.05 (for statistically nonsignificant) and “*” for *p* < 0.05 (for statistically significant) [131]. Reprinted with permission from Ref. [131]; Copyright 2019 National Academy of Sciences USA.

**Figure 16:**
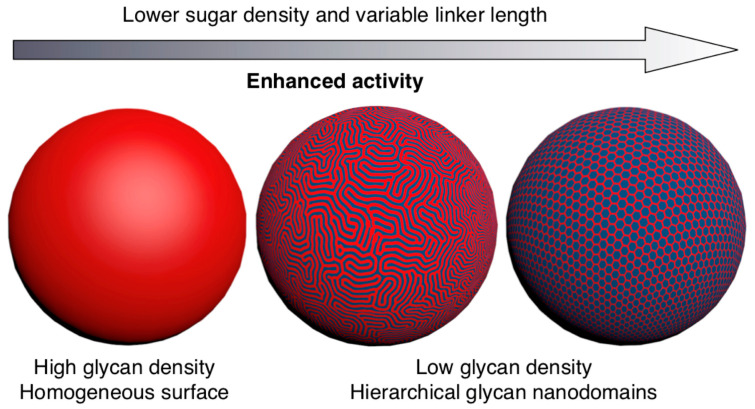
The effect of sugar density on linker length in a bicomponent cell-membrane mimic morphology [131]. Reproduced with permission from Ref. [131]; copyright 2019 National Academy of Sciences USA.

Figure 14 and Figure 15 outline in more details such an experiment in which the sequence and concentration of the carbohydrate Lactose was placed in a sequence-defined architecture on the periphery of the glycodendrimersome glycan (Figure 14). Figure 15 shows the unexpected dependence between the activity of binding and the concentration-sequence. Surprisingly, the lowest activity of binding was observed at the highest concentration of carbohydrates while the highest activity was observed at the lowest concentration in a sequence-defined arrangement. These results changed our way of thinking about the activity of the interaction of sugar binding proteins to concentration and sequence of sugars. This higher activity at lower concentration can be explained by a different rate contact that depends on concentration, rather than a constant rate constant that changes rate as a function of concentration and this event can be explained only by a different morphology of the surface of the glycan that like in the case of block copolymers is concentration dependent. Figure 16 shows the dependence of the glycan surface morphology of sequence and concentration that is responsible for the increase in reactivity at low sugar concentration.

## 9. Hybrids of Dendrimersomes/Glycodendrimersomes with Bacteria and Human Cell Membranes and Dendrimersomes Engulf Living Bacteria via Endocytosis

**Figure 17:**
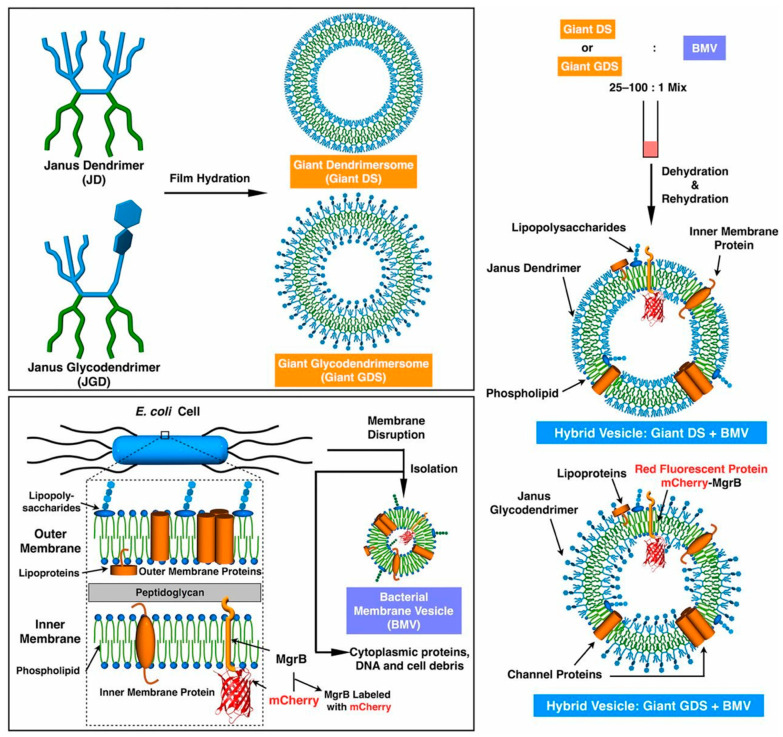
Illustration for the preparation and coassembly of hybrid giant vesicles from giant DSs, giant GDSs, and *E. coli* BMVs enriched with mCherry-MgrB [127]. Reprinted with permission from Ref. [127]; Copyright 2016 National Academy of Sciences USA.

Hybrid dendrimersomes/glycodendrimersomes with bacteria and human cell membranes were successfully co-assembled to transfer many of the components of the natural cell membranes in the resulting hybrid. This is a very important accomplishment since incorporation even of natural transmembrane proteins into synthetic cell membranes is a very complex experiment. Figure 17 illustrates the co-assembly of bacterial cell membranes with glycodendrimersomes [113,127].

**Figure 18:**
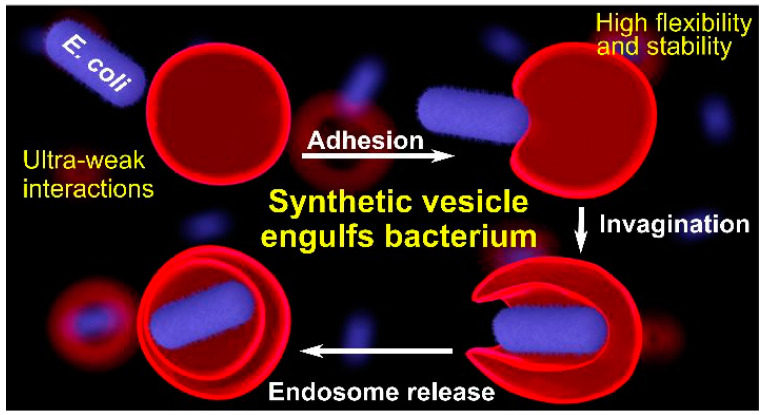
Engulfing living bacteria in dendrimersomes via endocytosis [116]. Reprinted with permission from Ref. [116]; Copyright 2019 American Chemical Society.

Figure 18 shows how a dendrimersome engulfs a living bacteria that stays alive and fights the dendrimersome wall in order to escape. This process can be visualized best by the movies available in the original publication [116]. We recommend the reader to consult these movies. These experiments can have numerous practical applications and demonstrate the excellent mechanical properties and stability of dendrimersomes and glycodendrimersomes.

## 10. Divergent Synthesis of Dendritic Macromolecules from Commercial Methyl Methacylate by DTC

**Figure 19:**
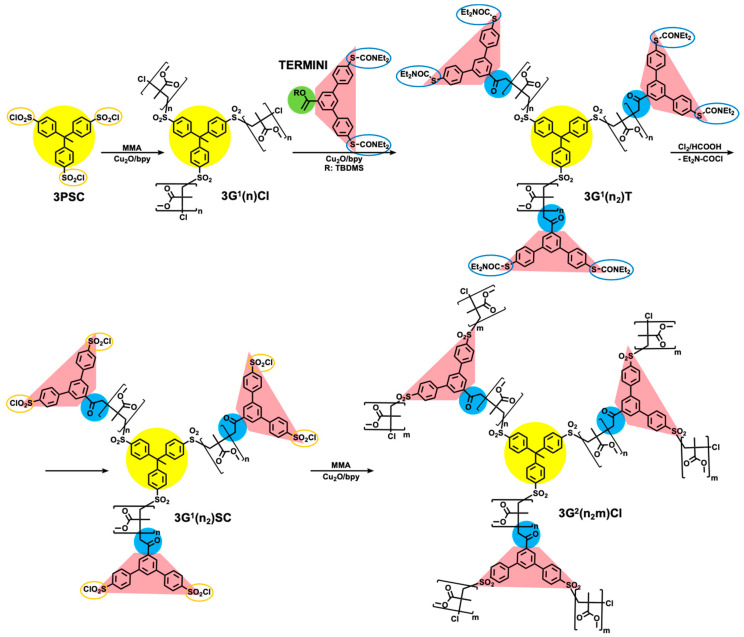
Divergent iterative synthetic strategy elaborated for synthesis of dendritic PMMA by a combination of LRP and TDC [17]. Reproduced with permission from Ref. [17]; Copyright 2003 American Chemical Society.

**Figure 20:**
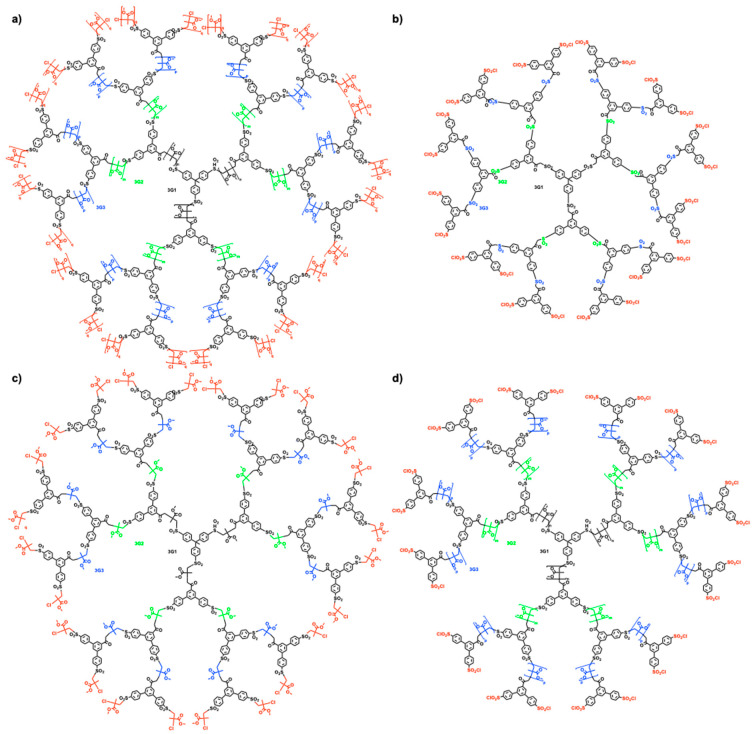
Dendritic macromolecules accessible by the combination of LRP of MMA and the bifunctional TERMINI starting from the 3PSC–trifunctional iniatiator:  (**a**) containing various DP of the PMMA per arm and PMMA chain ends; (**b**) containing DP of PMMA equal to zero and sulfonyl chloride chain ends; (**c**) containing DP of PMMA equal to 1 and MMA adduct as chain ends; (**d**) containing various DP of the PMMA per arm and sulfonyl chloride as chain ends [17]. Reproduced with permission from Ref. [17]; Copyright 2003 American Chemical Society.

**Figure 21:**
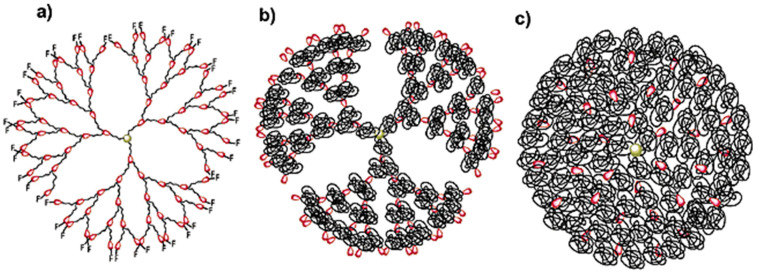
Depictions of dendritic macromolecules containing (**a**) small DP of PMMA to provide short and stiff chains between branching points, (**b**) medium DP of PMMA to provide flexible random coil conformation between the branching points, and (**c**) large DP of PMMA to provide long entangled chains between branching points. F denotes a functional group [17]. Reprinted with permission from Ref. [17]; Copyright 2003 American Chemical Society.

The TDC methodology was highlighted in Figure 3. Naming this methodology ‘click chemistry’ after so many years was inspired by the approach of Sharpless to click chemistry from his pioneering paper from 2001 [6]. In his paper, he defined a series of old methodologies including the original Huisgen and interfacial amidation reactions as ‘click’ reactions. Therefore, we feel that it is reasonable to state that the TDC reaction outlined in Figure 3 belongs to the class of click reactions. The first application of TDC was reported in 2003 and provided access to the divergent synthesis of dendritic macromolecules from the commercial monomer methyl methacrylate [17]. We believe that this methodology applies to any commercial methacrylate or styrene monomer. The synthesis of a methyl methacrylate dendritic macromolecule by this methodology is outlined in Figure 19, while Figure 20 and Figure 21 show the unusual structures and three-dimensional architectures of the resulting dendritic macromolecules. This methodology was expanded to different multiplicities at the focal point and to simpler TERMINI molecules [30,31,32,135].

## 11. Organizing Frontiers in Macromolecular and Supramolecular Science Symposia together with Bogdan C. Simionescu

In 1981, one of us (VP) defected the country he was born and educated. Political changes allowed him to return to Romania in 1995, accompanied by a small group of scientists and a former Ph.D. student.

**Figure 22:**
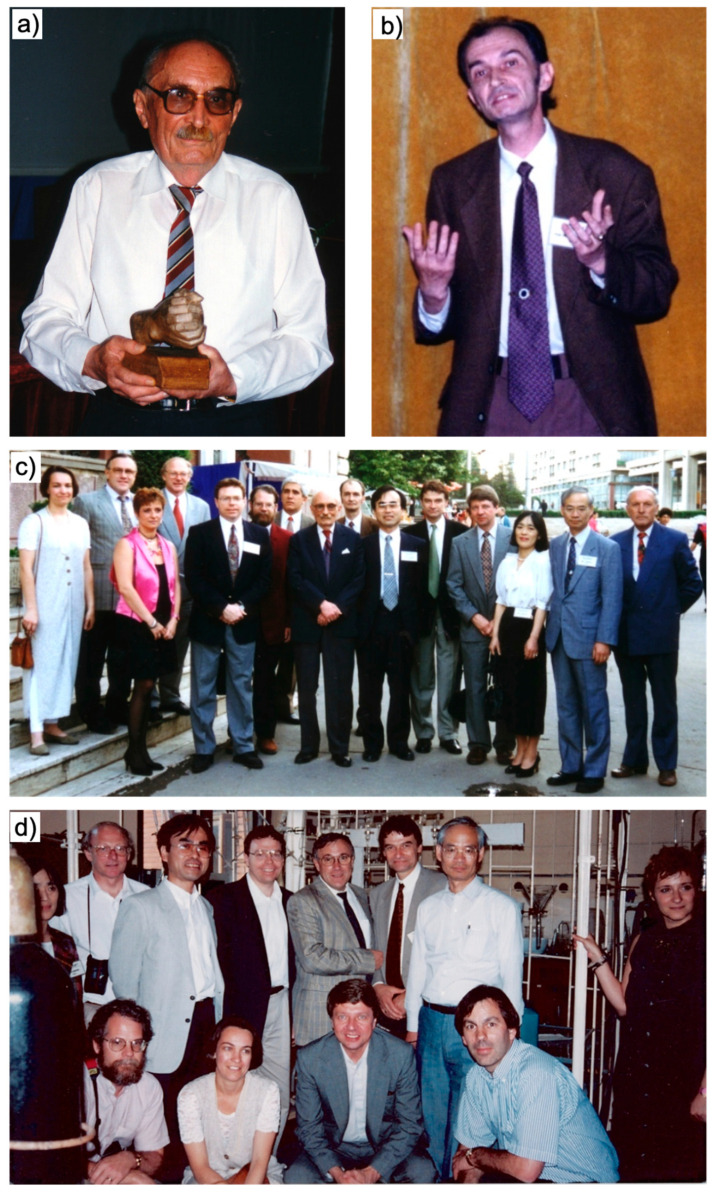
(**a**) The 1995 symposium celebrating the 75th anniversary of professor Cristofor I. Simionescu; (**b**) Bogdan Simionescu presenting his lecture during the 1995 symposium; (**c**) Professor C.I. Simionescu together with the lectures of the 1995 symposium; (**d**) the lecturers of the 1995 symposium visiting V. Percec former laboratory.

**Figure 23:**
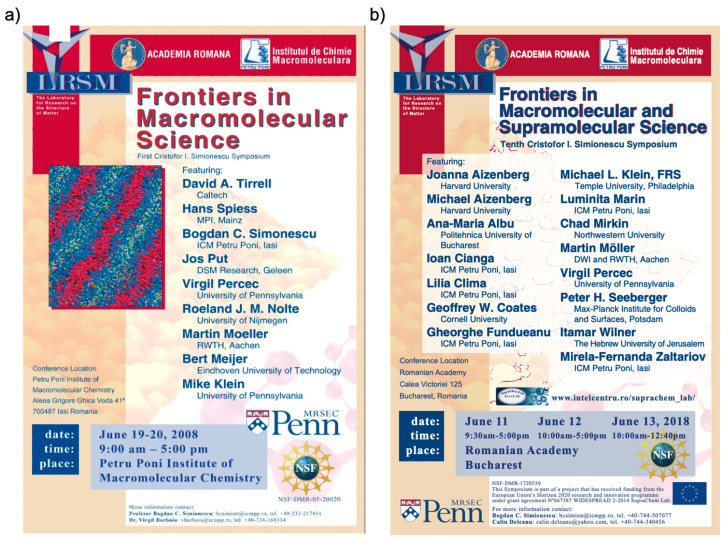
Posters of 1st and 10th Frontiers in Macromolecular and Supramolecular Science Symposia.

**Figure 24:**
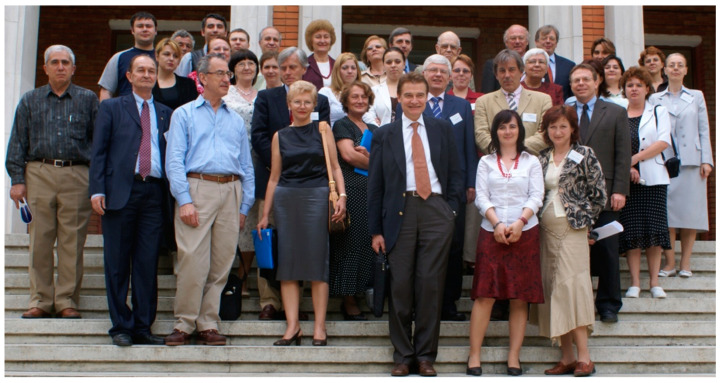
Lectures and participants of the first Frontiers in Macromolecular and Supramolecular Science Symposium.

The short visit was used to celebrate the 75th anniversary of his Ph.D. mentor Professor Cristofor I. Simionescu with a scientific symposium. This visit created life-time friendship with all the speakers at this symposium. Only one of the speakers at this symposium did not come from abroad. His name was Bogdan C. Simionescu. Few photos from this symposium are shown in Figure 22. This symposium also allowed me to visit with all other speakers for the first time in many years the laboratory in which I (VP) conducted the research for my Ph.D. thesis (Figure 22). In 2008, when Professor Cristofor I. Simionescu was no longer with us, I convinced Bogdan Simionescu to start duplicating the 1995 symposium and dedicate it to the memory of his father. Since I had organized previously numerous Gordon Research Conferences and other international symposia, the decision was made to maintain the format of the 1995 symposium with less than 10 invited lecturers and no other speakers. This would provide sufficient time for the young generation of scientists to discuss with the invited speakers. The poster of the 2008 symposium entitles “Frontiers in Macromolecular and Supramolecular Science”, First Cristofor I. Simionescu Symposium is shown in Figure 23 together with the poster of the 10th and last symposium. Most of the lectures from the 1995 symposium attended also the 2008 symposium, and this rotation of some of the same speakers was maintained for all 10 Symposia. A photo of the participants and lecturers at the First Frontiers in Macromoleculer and Supramolecular Science Symposium from 2008 on the stairs of the Institute in Iasi is shown in Figure 24. All 10 Symposia started to take place both at the Academy in Bucharest and at the “Petru Poni” Institute of Macromolecular Chemistry in Iasi. I insisted that we would maintain the original arrangement of the 1995 symposium that was co-organized, in fact behind the scene, by the person we were celebrating, Cristofor I. Simionescu. In order to recover the difference of time for lecturers from Japan and United States, as Professor Cristofor I. Simionescu organized it in 1995, all speakers would spend one or two days in Sinaia visiting the surroundings, including Peles and Bran Castles, as well as Brasov. This would blend history with culture and science.

Each symposium would end with a short visit to the Monasteries of Bucovina where both Professor Cristofor I. Simionescu and myself (VP) were born. This was a remarkable series of symposia that could be organized only by a very special collaboration, friendship, and respect towards the father of Bogdan Simionescu by both Bogdan and by one of the former students of his father (VP). These symposia will never be duplicated again in any place and by anybody else. However, the desire to restart a similar series by the same concept exists in the mind of all previous speakers and organizers of the first 10 Frontiers in Macromolecular and Supramolecular Science Symposia.

## 12. Conclusions

This perspective discusses a very narrow topic of research from our laboratories. It does not even touch related topics of biological relevance such as mimics of transmembrane protein mimics, Tobacco Mosaic Virus [136,137,138,139,140,141,142], Frank–Kasper phases that are available also in lipids [143,144,145,146,147,148,149,150], new synthetic methods-based on Ni rather than Pd-catalyzed and other reactions [151,152,153,154], or additional improvements of living radical polymerizations expanding on the 1995 publication on arenesulfonyl chloride initiators [155,156,157]. Not even the work on helical chirality pioneered during my graduate studies in the laboratory of C. I. Simionescu [158] was discussed here. We would like to mention that the amphiphilic Janus dendrimers and glycodendrimers dicussed briefly here are precursors to the one-component multifunctional sequence-defined ionizable amphiphilic Janus dendrimers (IAJDs) elaborated by our laboratory for the targeted delivery of mRNA [159,160,161]. The goal of this perspective is to make the synthetic community adopt the click concept of Sharpless, expand it to many other organic reactions, hoping that one day we will teach undergraduate and graduate organic chemistry only with click reactions.

## Data Availability

Not Applicable.
